# Immunological Challenges and Emerging Strategies in Dengue Virus Vaccine Development

**DOI:** 10.3390/vaccines14100902

**Published:** 2026-10-15

**Authors:** Xuelian Ma, Mengyuan Zhao, Yaqun Xu, Yinghan Jiang, Yang Yang, Luping Lei, Minghui Yang

**Affiliations:** 1 School of Medical Engineering, School of Interdisciplinary Science, Affiliated Zhuhai People’s Hospital, Beijing Institute of Technology, Zhuhai 519088, China; 2 National Clinical Research Center for Infectious Disease, Shenzhen Third People’s Hospital, Second Hospital Affiliated to Southern University of Science and Technology, Shenzhen 518112, China; young@mail.sustech.edu.cn; 3 School of Life Science, School of Interdisciplinary Science, Beijing Key Laboratory of Mucosal Immunity Vaccines Against Respiratory Viruses, Beijing Key Laboratory of Intelligent Precision Gene Therapy R&D and Translation, Key Laboratory of Molecular Medicine and Biotherapy, Key Laboratory of Medical Molecule Science and Pharmaceutics Engineering, Beijing Institute of Technology, Beijing 100081, China; 4 Department of General Practice, Beijing Tongren Hospital, Capital Medical University, Beijing 100730, China

**Keywords:** dengue virus, dengue vaccine, antibody-dependent enhancement, mRNA vaccine

## Abstract

Dengue is one of the most rapidly spreading arboviral diseases worldwide, and the development of effective vaccines has long been constrained by antibody-dependent enhancement (ADE) and the difficulty of inducing protective immunity against multiple viral serotypes. Although several live-attenuated dengue vaccines have received regulatory approval in different jurisdictions, including Dengvaxia (CYD-TDV), Qdenga (TAK-003), and, more recently, Butantan-DV in Brazil, important challenges remain regarding vaccine safety in relation to pre-vaccination dengue serostatus, balanced cross-serotype protection, and the durability of vaccine-induced immunity. This review focuses on the immunopathogenic mechanisms of dengue virus infection, with particular emphasis on how ADE and immune bias impose critical constraints on vaccine design. We systematically summarize the technological platforms, immunological rationale, and major limitations of licensed and investigational dengue vaccines. Building on this analysis, the potential advantages and remaining challenges of emerging vaccine technologies, particularly mRNA vaccines, in precise antigen design, multivalent immune regulation, and safety optimization are further discussed. This review aims to provide a theoretical basis and strategic reference for the development of next-generation dengue vaccines with improved safety and efficacy.

## 1. Introduction

Dengue is an acute arboviral infectious disease caused by dengue virus (DENV) and transmitted primarily by *Aedes aegypti* and *Aedes albopictus* mosquitoes. It is widely endemic in tropical and subtropical regions. As one of the most rapidly spreading arboviral diseases worldwide, dengue currently threatens the health security of approximately 4 billion people in more than 100 countries and regions [1,2]. Epidemiological studies estimate that approximately 390 million DENV infections occur annually, of which about 96 million are clinically symptomatic. Severe cases may progress to dengue hemorrhagic fever (DHF) or dengue shock syndrome (DSS), resulting in a substantial mortality burden [3,4].

According to the World Health Organization (WHO), the number of reported dengue cases worldwide reached a record high in 2024, indicating a marked and sustained increase in the global disease burden [5]. In recent years, driven by multiple factors including global climate change and accelerated globalization, the geographic and ecological suitability of the dengue vectors *Aedes aegypti* and *Aedes albopictus* has expanded and increased substantially, thereby reshaping the transmission landscape of DENV [6,7,8]. In this context, dengue transmission is expanding beyond its traditional tropical and subtropical endemic areas into temperate regions. Increasing autochthonous transmission events have been reported in previously non-endemic areas, including Europe and North America, suggesting that dengue is evolving from a regionally endemic disease into a global public health concern [9,10].

The current epidemiological complexity of dengue is closely associated with the co-circulation of the four DENV serotypes. Phylogenetic evidence supports an ancestral sylvatic transmission cycle involving non-human primates and forest-associated Aedes mosquitoes, from which epidemic DENV lineages independently became established in sustained human transmission [11,12]. The expansion of urban transmission mediated by *Aedes aegypti*, together with increasing human mobility, facilitated DENV dissemination. During and after World War II, intensified movement of people and materials promoted multi-serotype circulation in Southeast Asia and the Pacific, contributing to hyperendemic transmission [12]. The resulting increase in heterotypic secondary infections created an epidemiological setting in which pre-existing cross-reactive antibodies could mediate antibody-dependent enhancement (ADE), thereby increasing the risk of severe dengue [13]. This pattern of multi-serotype endemicity is directly relevant to vaccine development because effective vaccines must induce balanced multitypic protection while minimizing enhancement-prone immune responses.

However, unlike the case of many other viral diseases, dengue vaccine development faces unique and complex immunological constraints. DENV consists of four antigenically distinct serotypes, DENV-1 to DENV-4, and cross-protective immunity among different serotypes is generally limited and transient [14]. More importantly, cross-reactive antibodies generated by previous infection or immunization may facilitate viral entry into Fc receptor-positive cells and enhance viral replication through antibody-dependent enhancement (ADE) during secondary infection with a heterologous serotype, thereby substantially increasing the risk of severe disease [15]. This phenomenon means that vaccine-induced immune responses may, under certain conditions, become pathogenic rather than protective, representing a central obstacle to dengue vaccine safety.

Against this immunological background, an ideal dengue vaccine should not only induce broad and durable neutralizing antibody responses against all four serotypes, but also avoid eliciting non-neutralizing or low-affinity antibodies that may mediate ADE [9,15]. However, existing evidence indicates that both natural infection and vaccination often generate serotype-biased and asymmetric immune responses [14,16], further complicating vaccine design. Therefore, the central challenge in dengue vaccine development is not simply whether an immune response can be induced, but how to achieve immune regulation that is both safe and protective. To date, several live-attenuated dengue vaccines have received regulatory approval in different jurisdictions, including CYD-TDV, TAK-003, and, more recently, Butantan-DV in Brazil. However, their regulatory indications, programmatic recommendations, and availability vary substantially across regions. Important limitations remain regarding safety in specific populations, the breadth and balance of protection across DENV serotypes, and the long-term durability of vaccine-induced immunity [17,18]. These differences in clinical performance may reflect not only the complex immunopathogenesis of dengue but also differences in vaccine design and antigenic composition. In particular, the extent to which individual vaccine platforms present DENV structural and non-structural antigens may influence the breadth and quality of humoral and virus-specific cellular immune responses. Accordingly, dengue vaccine performance should be evaluated not only in terms of neutralizing antibody responses, but also with consideration of serotype balance, cellular immunity, baseline serostatus, and vaccine antigenic architecture [19,20].

In the context of these persistent limitations, new technological approaches are urgently needed to enable more precise regulation of dengue-specific immune responses. With advances in molecular immunology and vaccine engineering, emerging vaccine platforms represented by mRNA vaccines provide new opportunities for dengue vaccine development. Through precise antigen design and controllable multivalent antigen expression, these platforms may optimize the quality of immune responses at the molecular level, thereby improving protective efficacy while potentially reducing ADE-associated risks [21,22]. Against this background, this review focuses on the immunopathogenic mechanisms of DENV infection, with particular emphasis on the key constraints imposed by ADE and immune bias on vaccine design. We systematically summarize the technological strategies and limitations of licensed and investigational dengue vaccines, and further discuss the potential advantages and challenges of mRNA vaccines in overcoming these immunological bottlenecks. This review aims to provide a theoretical basis and strategic reference for the development of next-generation dengue vaccines.

## 2. Immunopathogenic Features of Dengue Virus Relevant to Vaccine Design

DENV is a mosquito-borne, positive-sense, single-stranded RNA virus belonging to the genus *Flavivirus* within the family Flaviviridae. The biological characteristics of DENV and the complex host immune responses it elicits collectively determine the diversity of clinical manifestations and the risk of severe disease [23,24]. A systematic understanding of viral structure, replication, and pathogenic mechanisms provides the theoretical basis for vaccine development and the design of immunological intervention strategies.

### 2.1. Antigenic Architecture and Serotype Diversity Relevant to Vaccine Design

**Figure 1:**
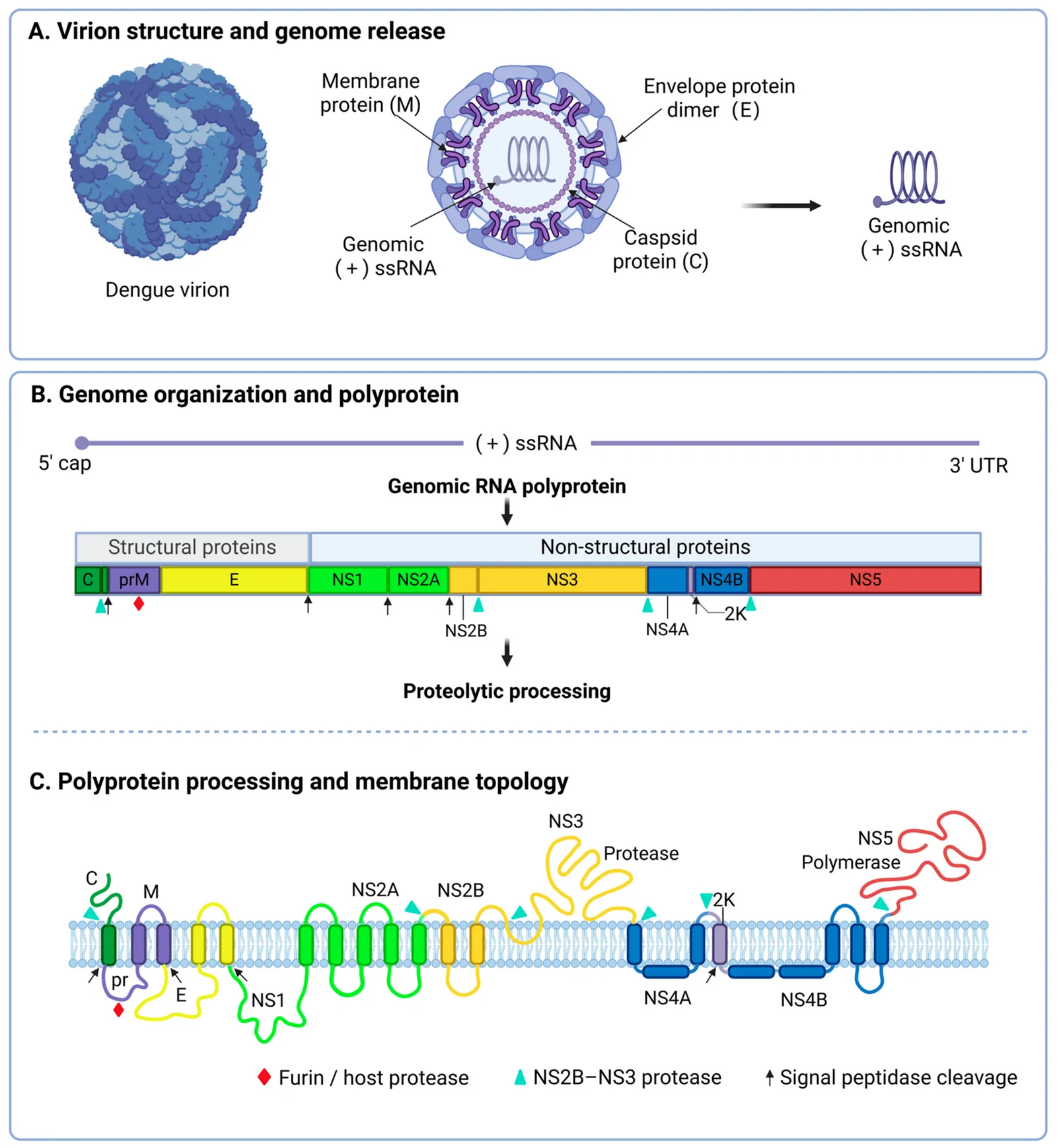
DENV virion and genome organization relevant to vaccine antigen selection. (**A**) Schematic organization of the mature DENV virion, highlighting the structural proteins C, prM/M, and E. (**B**) Genome organization and polyprotein processing showing the generation of structural and non-structural proteins. (**C**) Membrane-associated organization of the viral polyprotein. The figure emphasizes the distinction between structural antigens, particularly E as a major target of neutralizing antibodies, and non-structural antigens that contribute to viral replication and antiviral cellular immunity. Abbreviations: DENV, dengue virus; E, envelope protein; M, membrane protein; NS, non-structural protein; prM, precursor membrane protein; ssRNA, single-stranded RNA.

DENV is an enveloped positive-sense RNA virus whose single open reading frame encodes three structural proteins (C, prM/M, and E) and seven non-structural proteins (NS1, NS2A, NS2B, NS3, NS4A, NS4B, and NS5) [25]. From a vaccine-design perspective, the distinction between structural and non-structural antigens is particularly important. The E protein is the principal target of neutralizing antibodies, whereas several non-structural proteins contribute substantially to antiviral cellular immunity and, in the case of NS1, to both dengue pathogenesis and potentially protective immune responses. The E protein consists of three structural domains, EDI, EDII, and EDIII. Among them, EDII contains the fusion loop epitope (FLE), which is a highly conserved region across different serotypes and serves as an important antibody-binding site [26]. However, antibodies targeting the FLE are often broadly cross-reactive but weakly neutralizing, and under specific conditions may contribute to ADE, potentially increasing disease severity [27]. These vaccine-relevant antigenic features are summarized in Figure 1, which highlights the distinction between structural and non-structural DENV antigens.

Beyond its antigenic architecture, the cellular tropism of DENV also shapes the host immune response and disease pathogenesis. DENV primarily infects dendritic cells and cells of the monocyte–macrophage lineage. This tropism for immune cells provides an important biological basis for viral modulation of host immunity and ADE-associated pathogenic mechanisms [23,24,28].

DENV comprises four antigenically distinct serotypes, DENV-1 to DENV-4, and this serotype diversity represents a central challenge for vaccine development. Primary infection generally induces durable protection against the homologous serotype but only transient and incomplete cross-protection against heterologous serotypes [14]. After this cross-protection wanes, cross-reactive but insufficiently neutralizing antibodies may mediate ADE, thereby increasing the risk of severe disease during secondary heterologous infection. Meanwhile, original antigenic sin may further shape immune responses and contribute to suboptimal protection or immunopathological outcomes [15,16]. These immunological features determine that an ideal dengue vaccine must induce broad and durable protective immune responses against all four serotypes simultaneously. Achieving broad and effective tetravalent protection remains a central challenge in dengue vaccine development.

### 2.2. Host Immune Responses and Mechanisms of Immunopathogenesis

Host immune responses play a dual role in DENV infection. On the one hand, they mediate viral clearance and provide protective immunity; on the other hand, under specific conditions, they may contribute to immunopathogenesis and severe disease.

#### 2.2.1. Innate Immune Responses and Immune Evasion

Following DENV infection, innate antiviral responses centered on type I interferon (IFN-I) are the first to be activated. However, the virus evades immune clearance through multiple mechanisms. For instance, the NS5 protein can mediate STAT2 degradation, thereby inhibiting IFN signal transduction and weakening antiviral responses. The NS2B/NS3 protease complex can also target key host factors and interfere with innate immune recognition pathways [29,30]. In addition, DENV can modulate host autophagy to promote the formation of viral replication complexes, and enhance viral spread and immune evasion by inducing metabolic reprogramming and exploiting exosome-mediated pathways [31,32,33].

#### 2.2.2. Humoral Immune Responses and Antibody-Dependent Enhancement

In adaptive immunity, antibody responses can provide protection but may also mediate pathogenic effects. ADE is considered an important immunological mechanism contributing to severe dengue during secondary infection, although it does not fully explain all cases of severe disease. Cross-reactive non-neutralizing antibodies generated by prior infection can form immune complexes and mediate viral entry into monocytes through Fcγ receptors, thereby enhancing viral replication [15,34]. This mechanism was proposed by Halstead and has been supported by extensive experimental and epidemiological evidence [35,36].

According to the stage at which enhancement occurs, ADE can be classified into “extrinsic ADE” and “intrinsic ADE”. Extrinsic ADE facilitates viral entry into Fcγ receptor-positive cells through antibody-mediated uptake, whereas intrinsic ADE occurs after viral entry and promotes intracellular viral replication by altering antiviral immune responses [37]. During intrinsic ADE, enhanced viral replication has been associated with impaired type I interferon signaling and reduced antiviral gene expression, including disruption of the IFN-I/JAK-STAT pathway and decreased interferon-stimulated gene responses. These changes may weaken early antiviral defense and create an immune environment favorable for viral persistence and replication [38,39].

It should be noted that although ADE is considered an important contributor to severe dengue, it is a context-dependent phenomenon and does not fully explain all severe cases. With four antigenically distinct DENV serotypes, 12 ordered heterotypic primary–secondary infection sequences are theoretically possible. Severe dengue has been documented following a broad range of these heterotypic sequences; in a large surveillance analysis in Thailand, 11 of the 12 possible sequential serotype combinations were observed among individuals who developed dengue hemorrhagic fever during the second detected infection, with DENV-4 followed by DENV-3 being the only sequence not observed in that dataset [40]. Importantly, however, the occurrence of severe disease across multiple heterotypic sequences does not imply equivalent disease risk among them. Recent epidemiological and antigenic analyses indicate that disease risk varies according to the order of infecting serotypes and the antigenic distance between the primary and secondary viruses [41]. The magnitude and functional quality of pre-existing antibodies, including their epitope specificity and neutralizing capacity, may further modulate the outcome of secondary infection [42,43]. These findings underscore the multifactorial nature of severe dengue and suggest that ADE-related risk is shaped by the interaction of viral antigenic relationships, antibody characteristics, and host factors.

#### 2.2.3. Cellular Immune Responses and Immune Bias

In addition to humoral immunity, T cells also play important roles in DENV infection. During primary infection, CD8^+^ T cells can effectively clear infected cells. However, during secondary infection, cross-reactive memory T cells may exhibit inefficient or biased responses, a phenomenon referred to as original antigenic sin. This may lead to excessive release of inflammatory cytokines while limiting viral clearance, thereby aggravating immunopathological injury and promotingvascular permeability [16,37].

#### 2.2.4. Immunopathogenic Effects and Constraints on Vaccine Design

Severe dengue is characterized by plasma leakage and vascular dysfunction resulting from interactions between viral factors and host responses that disrupt vascular barrier integrity [44,45,46]. Recent evidence has identified dengue virus non-structural protein 1 (NS1) as a critical viral factor contributing to endothelial dysfunction and vascular leakage. Secreted NS1 directly affects endothelial cells and disrupts the endothelial glycocalyx, resulting in increased vascular permeability [47,48,49]. NS1-induced inflammatory responses may further aggravate endothelial injury. Anti-NS1 antibodies may also contribute to disease pathology through molecular mimicry and immune-mediated cellular injury, including thrombocytopenia [50]. Therefore, severe dengue vascular dysfunction results from both NS1-mediated endothelial injury and immune-mediated damage rather than inflammation alone.

**Figure 2:**
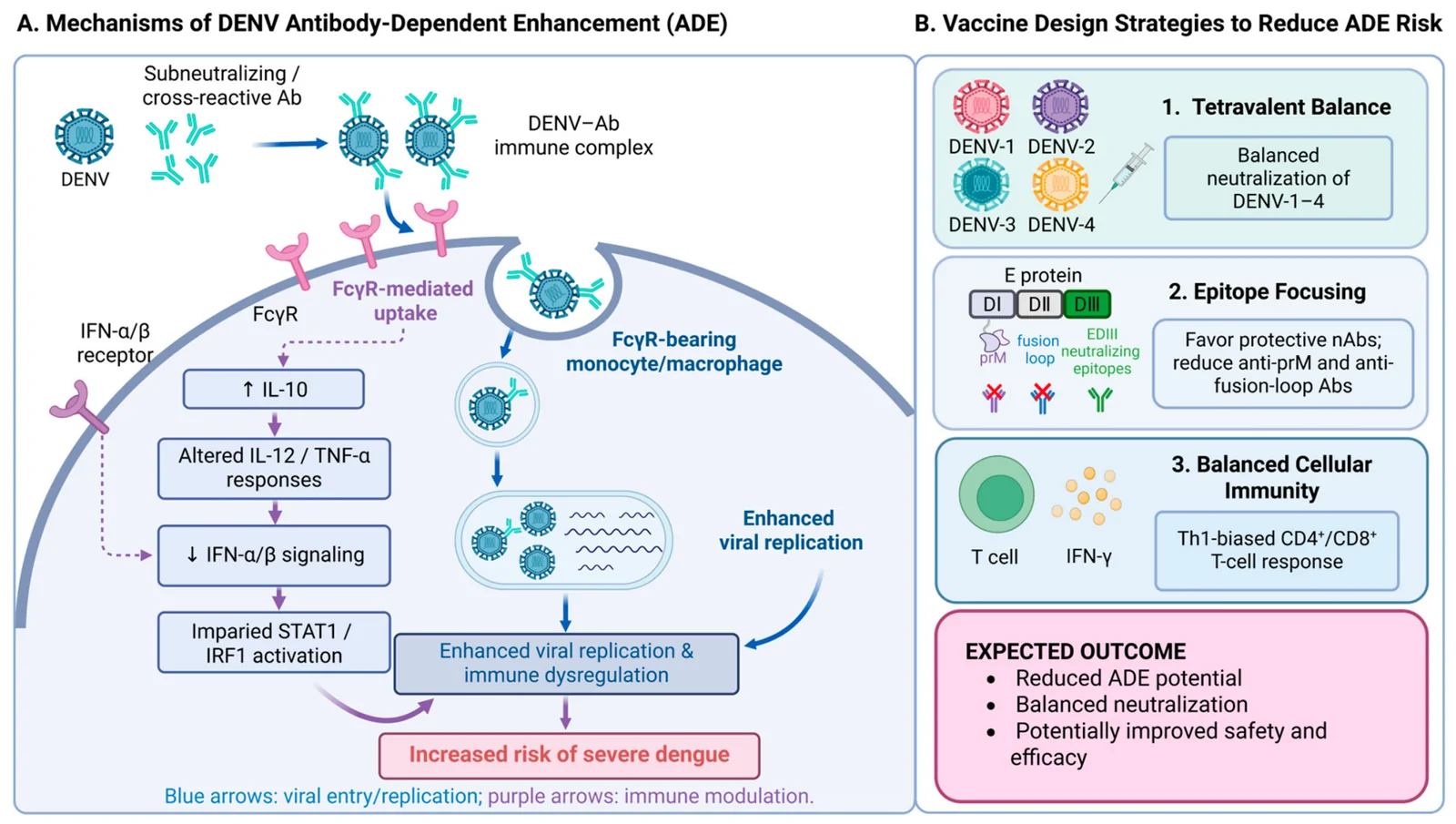
Mechanisms of dengue virus antibody-dependent enhancement and vaccine design strategies to mitigate ADE. (**A**) Schematic overview of dengue virus (DENV) antibody-dependent enhancement (ADE). Subneutralizing or cross-reactive antibodies bind DENV particles and form DENV–antibody immune complexes, which are internalized by Fcγ receptor (FcγR)-expressing monocytes/macrophages through FcγR-mediated uptake. This process facilitates viral entry and intracellular replication. ADE-associated infection may also modulate innate immune signaling, including increased IL-10 production, altered IL-12/TNF-α responses, reduced type I interferon signaling, and impaired STAT1/IRF1 activation. These events collectively promote viral replication, immune dysregulation, and an increased risk of severe dengue. Blue arrows indicate viral entry and replication, whereas purple arrows indicate immune-modulatory pathways. Upward and downward arrows indicate increased and decreased expression or signaling activity, respectively. (**B**) Vaccine design strategies to reduce ADE potential. Broad tetravalent protective immunity aims to induce comparable neutralizing responses against DENV-1 to DENV-4. Epitope-focused antigen design may enhance protective neutralizing antibody responses while limiting antibodies against ADE-associated epitopes, such as prM and the envelope fusion loop. Effective cellular immunity, particularly Th1-biased CD4^+^ and CD8^+^ T-cell responses, may further support antiviral protection. Together, these strategies may reduce ADE potential, improve serotype-balanced neutralization, and enhance vaccine safety and efficacy. Abbreviations: Ab, antibody; ADE, antibody-dependent enhancement; DENV, dengue virus; FcγR, Fc gamma receptor; IFN, interferon; IL, interleukin; IRF1, interferon regulatory factor 1; nAbs, neutralizing antibodies; prM, precursor membrane protein; STAT1, signal transducer and activator of transcription 1; TNF-α, tumor necrosis factor alpha.

In summary, the development of severe DENV-associated disease results from severe DENV-associated disease results from interactions between viral factors and protective and pathogenic immune responses [51]. These mechanisms indicate that inducing an immune response alone is insufficient to achieve protection. Instead, the quality and characteristics of the immune response play a decisive role in disease outcomes. This imposes critical constraints on vaccine design: effective immune protection must be induced while avoiding immune-mediated pathogenic effects. The major mechanisms underlying ADE and the corresponding vaccine design strategies aimed at minimizing ADE risk are summarized in Figure 2.

## 3. Regulatory Status, Immunological Characteristics, and Limitations of Approved Dengue Vaccines

The development of dengue vaccines has long been constrained by the complex immunopathogenic mechanisms of dengue virus infection. Unlike most viral diseases, protective immunity and pathogenic immunity in DENV infection substantially overlap. Therefore, a dengue vaccine must not only induce broadly effective neutralizing antibodies, but also avoid eliciting non-neutralizing antibody responses that may mediate ADE [52,53]. In addition, antigenic differences and cross-reactivity among the four serotypes further increase the complexity of immune regulation.

### 3.1. Key Immunological Constraints on Vaccine Design

An ideal dengue vaccine must achieve a delicate balance among immune breadth, potency, and safety. First, it should induce broad and durable neutralizing antibody responses against all four serotypes, thereby reducing the risk of secondary infection. Second, it should avoid generating low-affinity cross-reactive antibodies to minimize the possibility of ADE [54]. However, studies have shown that antibody responses after dengue vaccination often exhibit “serotype bias”, in which a dominant immune response is generated against one serotype, while responses to other serotypes remain relatively weak [23,55].

In addition to neutralizing antibodies, vaccine-induced cellular immunity is an important component of protective immunity against DENV. T-cell responses contribute to viral clearance, immune memory, and the quality of long-term protection. Importantly, the breadth of cellular immunity may be influenced by the antigenic repertoire presented by a vaccine. Replication-competent live-attenuated flavivirus vaccines, such as YF-17D and the Japanese encephalitis vaccine SA14-14-2, retain the capacity to express both structural and non-structural viral proteins and can therefore present a broad range of viral antigens to the immune system. Consistent with this principle, YF-17D vaccination induces broad and durable T-cell responses recognizing both structural and non-structural proteins, while virus-specific cellular immune responses have also been demonstrated following SA14-14-2 vaccination [56,57]. In contrast, in chimeric dengue vaccines, the origin and breadth of non-structural antigens are determined by the viral backbone. Because DENV non-structural proteins constitute important targets of virus-specific T-cell responses, differences in their representation may influence the breadth and quality of vaccine-induced cellular immunity and should therefore be considered together with neutralizing antibody responses when evaluating vaccine performance [58].

### 3.2. Clinical Performance and Current Regulatory Status of Approved Dengue Vaccines

#### 3.2.1. CYD-TDV (Dengvaxia)

CYD-TDV (Dengvaxia) was the first licensed dengue vaccine. It is a tetravalent live-attenuated chimeric vaccine constructed on theYF-17D backbone, in which the prM and E proteins are derived from DENV-1 to DENV-4, whereas the non-structural proteins are derived from YF-17D [59]. Thus, CYD-TDV presents DENV structural antigens capable of inducing DENV-specific antibody responses but does not contain DENV-derived non-structural proteins. Because DENV non-structural proteins contain important targets of virus-specific T-cell responses, this antigenic architecture may limit the breadth of de novo DENV non-structural protein-specific cellular immunity. Consistent with this design, CYD-TDV has been shown to induce prominent YF-17D NS3-specific CD8^+^ T-cell responses together with DENV serotype-specific helper T-cell responses [60]. Clinical studies have shown that CYD-TDV provides moderate protection in individuals with prior DENV infection. However, in seronegative individuals, vaccination is associated with an increased risk of severe dengue during subsequent natural infection, a phenomenon that has been interpreted in the context of ADE-related mechanisms [17]. The large-scale vaccination campaign in the Philippines in 2017, involving more than 800,000 children, subsequently became the focus of major public controversy following reports of deaths among vaccine recipients and concerns regarding CYD-TDV use in dengue-seronegative individuals [61]. The current regulatory status and indications of CYD-TDV vary across jurisdictions. In the United States, the vaccine is approved for individuals aged 6–16 years with laboratory-confirmed previous dengue infection who live in endemic areas. In the European Union, its marketing authorization was withdrawn in October 2025 at the request of the marketing authorization holder for commercial reasons. Therefore, the current use of CYD-TDV should be interpreted within jurisdiction-specific regulatory and public-health frameworks rather than as a uniform global recommendation.

From an immunological perspective, the absence of DENV-derived non-structural antigens and the resulting limitation in de novo DENV non-structural protein-specific cellular immunity have been proposed as potential factors contributing to the serostatus-dependent performance of CYD-TDV [62]. In dengue-seronegative recipients, vaccination may immunologically prime the host so that subsequent natural DENV infection occurs in the presence of pre-existing but insufficiently protective antibodies, potentially facilitating ADE-related responses and increasing the risk of severe disease [17,63]. However, the absence of DENV-derived non-structural proteins should not be considered the sole established mechanism underlying this outcome, which is likely to be multifactorial. In addition, CYD-TDV induces a markedly serotype-biased antibody response, with uneven immune intensity among different serotypes. This immune asymmetry further increases the likelihood of unfavorable immune responses during heterologous infection [64].

#### 3.2.2. TAK-003 (Qdenga)

TAK-003 (Qdenga), is a tetravalent live-attenuated vaccine based on the attenuated DENV-2 PDK-53 backbone, with the DENV-1, DENV-3, and DENV-4 components expressing their respective prM and E proteins on this backbone [65] Unlike CYD-TDV, TAK-003 contains DENV non-structural proteins, although these are derived from DENV-2; vaccination can nevertheless induce T-cell responses to DENV-2 non-structural proteins as well as cross-reactive responses to the other DENV serotypes [66].

Results from the phase III TIDES trial (DEN-301) showed that, within 12 months after vaccination in children and adolescents aged 4–16 years, the overall protective efficacy of TAK-003 reached 80.2%. Protection against severe dengue and dengue-related hospitalization was also observed during follow-up. The vaccine also demonstrated substantial protection in dengue-seronegative participants, although efficacy varied according to serotype and baseline serostatus [18]. These findings suggest that optimizing the vaccine backbone and antigen expression strategy can reduce ADE risk to some extent.

However, with extended follow-up, the protective efficacy of TAK-003 against different serotypes showed significant variation. Final 4.5-year follow-up data demonstrated substantial serotype-dependent differences in vaccine efficacy. In dengue-seronegative participants, protection was maintained mainly against DENV-1 and DENV-2, whereas protection against DENV-3 was not demonstrated and the limited number of DENV-4 cases prevented definitive conclusions [67]. Although TAK-003 contains DENV-derived non-structural proteins and induces cross-reactive cellular immunity, its antigenic composition remains centered on the DENV-2 backbone, and balanced protection against all four serotypes, particularly in dengue-seronegative individuals, requires further evaluation.

#### 3.2.3. Butantan-DV

More recently, Butantan-DV, a single-dose live-attenuated tetravalent dengue vaccine, demonstrated favorable efficacy and safety profiles in a phase III trial involving participants aged 2–59 years, supporting its potential as a simplified dengue vaccination strategy [68]. Butantan-DV contains attenuated DENV-1, DENV-3, and DENV-4 components, whereas its DENV-2 component is chimeric, expressing DENV-2 prM/E proteins on an attenuated DENV-4 backbone. Consequently, the vaccine contains DENV-derived non-structural proteins from DENV-1, DENV-3, and DENV-4, but not from DENV-2, and therefore does not provide a fully serotype-matched non-structural protein repertoire across all four vaccine components [69]. Following regulatory approval in Brazil in late 2025 for individuals aged 12–59 years, Butantan-DV further expanded the landscape of licensed dengue vaccines [70]. Five-year follow-up of the phase III trial showed an overall efficacy of 65.0% against symptomatic virologically confirmed dengue, including 77.1% in dengue-experienced and 58.9% in dengue-naive participants. Serotype-specific efficacy was 73.0% against DENV-1 and 55.7% against DENV-2; however, efficacy against DENV-3 and DENV-4 could not be determined because no cases occurred during follow-up [71]. Although the single-dose regimen provides an important operational advantage, continued surveillance is required to define its long-term durability, safety, and breadth of protection.

Overall, currently available dengue vaccines differ substantially in their representation of DENV non-structural antigens. These differences highlight that a tetravalent formulation does not necessarily ensure equivalent serotype-matched immunity or balanced protection against all four DENV serotypes. However, the extent to which non-structural antigen composition directly determines clinical efficacy remains to be fully established. In particular, NS1 has emerged as a potential complementary antigen because of its dual roles in dengue pathogenesis and host immunity. Experimental studies suggest that NS1-specific immune responses may mitigate endothelial dysfunction and vascular leakage, supporting the incorporation of NS1-based strategies into next-generation dengue vaccine designs [72]. In addition, the durability of vaccine-induced immunity remains a major challenge. Evidence indicates that neutralizing antibody responses may decline over time, with heterogeneous kinetics among different DENV serotypes, potentially contributing to reduced protective immunity and limited long-term protection [73].

In summary, currently approved dengue vaccines have established the feasibility of vaccine-mediated protection against dengue but also revealed persistent challenges associated with baseline serostatus, serotype-specific efficacy, immune durability, and post-licensure safety. These limitations reflect the complexity of DENV immunopathogenesis and the difficulty of achieving broad, effective, and long-lasting protection against all four serotypes. Future vaccine development should therefore focus on rational antigen selection, optimization of humoral and cellular immunity, and the application of emerging technological platforms to generate safer and more durable tetravalent protection.

## 4. Investigational Dengue Vaccine Platforms and Their Responses to Core Challenges

Under the key constraints imposed by ADE and imbalanced tetravalent immunity, various vaccine platforms show clear differences in safety, immunogenicity, and controllability. The current development of investigational dengue vaccines can essentially be viewed as diverse strategies to address the central question of how to achieve balanced protection while avoiding ADE. At present, multiple candidate vaccines based on different technological platforms remain in clinical or preclinical development (Table 1).

### 4.1. NIAID TV003/TV005 and the Evolution of Live-Attenuated Tetravalent Vaccine Design

Live-attenuated vaccines can mimic the process of natural infection and induce robust humoral and cellular immune responses, making them one of the strategies closest to an ideal immune state. TV003/TV005, developed by the U.S. National Institute of Allergy and Infectious Diseases (NIAID) and advanced by the Butantan Institute, is a tetravalent live-attenuated vaccine platform. Its core design is based on 3′ untranslated region (3′ UTR) deletion mutations and chimeric construction strategies, aiming to induce balanced immunity against all four serotypes [74]. Studies have shown that a single dose can induce tetravalent neutralizing antibody responses in 74–92% of DENV-naive individuals for TV003 and approximately 90% for TV005. Upon re-vaccination, these vaccines exhibit features close to “sterilizing immunity”, suggesting strong immune memory [75].

TV005 significantly improves tetravalent immune balance by increasing the dose of the DENV-2 component, thereby overcoming the limitation of “dominant serotype bias” commonly observed in traditional live-attenuated vaccines [76]. Recent human challenge model studies have further confirmed that TV005 provides significant protection against DENV-2 and DENV-3, supporting its progression into later-stage clinical evaluation [77]. However, live-attenuated vaccines also have clear limitations. Differences in the in vivo replication capacity of viruses from different serotypes can easily lead to immune responses biased toward a particular serotype, resulting in serotype-biased immune responses. In addition, ADE potential in live-attenuated dengue vaccines may be influenced by factors such as serotype balance, antigen composition and dose, and recipient serostatus. Optimization of vaccine components may therefore help reduce this potential risk.

### 4.2. Recombinant Subunit Vaccines

Subunit vaccines select specific antigens to avoid risks associated with viral replication and therefore have favorable safety profiles. The E protein (particularly its domain III, EDIII), along with the prM protein and NS1 protein, are all important targets for subunit vaccine development [78]. Among them, EDIII is widely used in vaccine design because it is enriched in neutralizing epitopes, whereas NS1 may provide protection against vascular leakage by inducing antibody-mediated immunity targeting non-structural proteins. A representative example is V180, a recombinant E protein-based vaccine developed by Merck. This vaccine combines a recombinant subunit antigen with either AS01 or aluminum adjuvant. In phase I clinical studies, it showed a good safety profile and a certain degree of neutralizing antibody response. However, its overall immunogenicity remained lower than that of live-attenuated vaccines, and multiple doses were required to maintain antibody levels [79].

In recent years, structure-based design has also been introduced to optimize subunit vaccines. By stabilizing the conformation of the E protein or constructing multivalent fusion antigens, this approach may broaden neutralizing antibody responses and reduce ADE risk [80]. However, these vaccines usually have relatively weak immunogenicity and require multiple immunizations and adjuvant enhancement. In addition, because they lack viral replication, the induced cellular immune responses are relatively limited, which may affect long-term protection.

### 4.3. DNA Vaccines

DNA vaccines use plasmid vectors to express DENV antigens in vivo, such as prM/E or NS1 proteins, and can induce both humoral and cellular immune responses. The tetravalent dengue DNA vaccine (TVDV) developed by the U.S. Naval Medical Research Center (NMRC) is a representative candidate vaccine, encoding the prM/E genes of all four serotypes. In a phase I clinical trial, TVDV showed a favorable safety profile, but neutralizing antibody levels were low, and detectable immune responses were induced only in a subset of participants [81].

To enhance immunogenicity, researchers have used electroporation delivery and prime–boost strategies, such as DNA priming followed by viral vector or protein vaccine boosting, to markedly improve antibody levels and T-cell responses [82]. In addition, molecular adjuvants, such as IL-12-expressing plasmids, have been used to enhance Th1 immune responses. Although DNA vaccines have advantages in stability and manufacturing, insufficient immunogenicity in humans remains a key bottleneck limiting their further development.

### 4.4. Viral Vector Vaccines

Viral vector vaccines use replication-defective or attenuated viruses to deliver DENV antigens and can induce both humoral and cellular immune responses. Adenovirus-vectored vaccines are representative examples. By expressing E or prM/E antigens, they can induce strong neutralizing antibody responses and CD8^+^ T-cell responses in animal models [83]. However, this strategy still faces several challenges, including interference from pre-existing vector immunity, insufficient antigen composition, and imbalanced tetravalent immunity. Current research mainly focuses on optimizing vector systems, incorporating non-structural proteins to enhance cellular immunity, and improving the coordination of multi-serotype immune responses. Overall, viral vector vaccines have advantages in inducing cellular immunity, but their safety and immune-regulatory properties require further evaluation.

### 4.5. Virus-like Particle Vaccines

Virus-like particles (VLPs) are genome-free particles formed by the self-assembly of viral structural proteins. Their antigenic conformation resembles that of authentic viruses, giving them strong immunogenicity without infectivity. Recent studies have shown that an engineered tetravalent DENV-VLP vaccine with prM removed can induce broad neutralizing antibody responses against all four serotypes in animal models. Masking ADE-associated epitopes, such as prM and fusion loop epitopes (FLE), may reduce the generation of cross-reactive non-neutralizing antibodies that can form DENV-antibody complexes and facilitate enhanced viral infection, thereby decreasing ADE risk [84,85]. VLP vaccines represent an important direction for next-generation non-replicating dengue vaccines. However, their clinical efficacy and durability remain to be validated.

**Figure 3:**
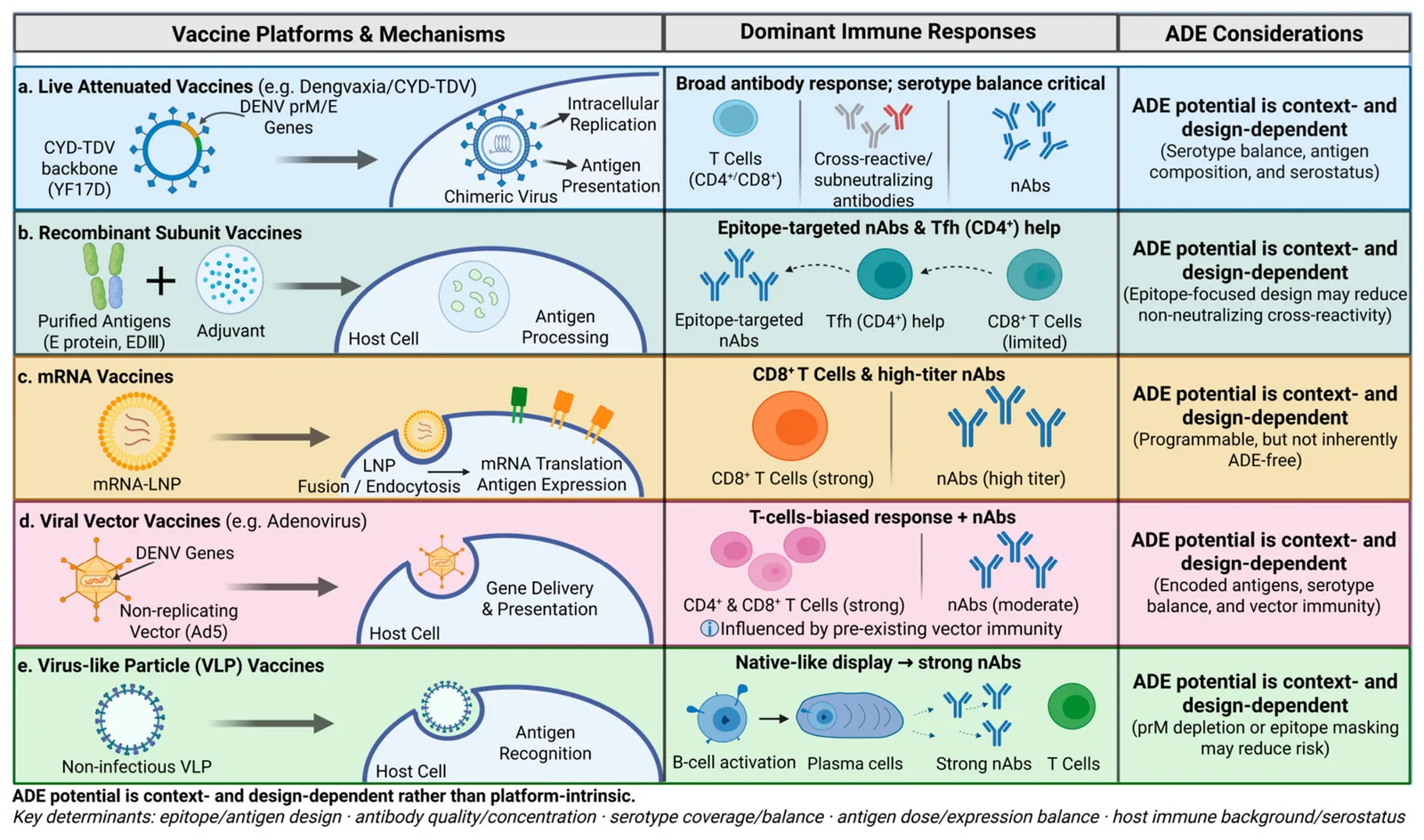
Dengue vaccine platforms, dominant immune responses, and context- and design-dependent ADE considerations. Dengue vaccine platforms, immune response profiles, and potential ADE risk implications. This schematic compares five major dengue vaccine platforms-live attenuated vaccines, recombinant subunit vaccines, mRNA vaccines, viral vector vaccines, and virus-like particle (VLP) vaccines-according to their antigen-delivery mechanisms, dominant immune responses, and potential implications for antibody-dependent enhancement (ADE). Live attenuated vaccines, such as CYD-TDV/Dengvaxia, can induce broad antibody and T-cell responses; however, ADE concern may be influenced by recipient serostatus and serotype-balanced immunity, particularly in seronegative individuals. Recombinant subunit vaccines may reduce ADE potential through epitope-focused antigen design that favors targeted neutralizing antibody responses while reducing cross-reactive non-neutralizing antibodies associated with ADE. mRNA vaccines enable intracellular antigen expression and may induce CD8^+^ T-cell responses together with high-titer neutralizing antibodies. Viral vector vaccines may elicit T-cell-biased immunity and moderate neutralizing antibody responses, with ADE risk depending on vector design, pre-existing immunity, and recipient serostatus. VLP vaccines mimic native virion-like antigen display and may promote B-cell activation and protective neutralizing antibody responses. Overall, ADE risk is context- and design-dependent rather than platform-intrinsic and is influenced by antibody characteristics, epitope/antigen design, antigen dose or expression, and host factors. Different colors represent the five vaccine platforms compared in this schematic. Arrows indicate antigen delivery processes and immune activation pathways.

Overall, although current investigational dengue vaccine platforms each offer distinct advantages, none has yet been able to simultaneously address the three major challenges of minimizing ADE, achieving broad tetravalent protective immunity, and maintaining durable long-term protection. As summarized in Figure 3, the major vaccine platforms differ substantially in their antigen-delivery strategies and immune-response profiles. However, ADE potential should not be regarded as an intrinsic property of any specific vaccine platform, but rather as a context- and design-dependent outcome influenced by antigen composition, epitope selection, antibody quality and concentration, serotype balance, antigen dose or expression balance, and host immune background. These limitations indicate that conventional vaccine approaches alone may be insufficient to achieve precise control over antigen expression and immune regulation, underscoring the need for more programmable and controllable platforms capable of simultaneously optimizing immunogenicity, tetravalent immune balance, and safety.

## 5. mRNA Vaccine Technology: A Programmable Platform for Mitigating ADE and Immune Imbalance

Compared with conventional vaccine technologies, mRNA vaccines offer a high degree of programmability in antigen design and immune regulation, providing a new technological pathway for addressing the core challenges in dengue vaccine development.

### 5.1. mRNA Vaccine Platforms: From Empirical Design to Precise Regulation

The COVID-19 pandemic drove a historic breakthrough in mRNA vaccine technology. The successful development and large-scale administration of the two mRNA vaccines BNT162b2 (Pfizer–BioNTech) and mRNA-1273 (Moderna) fully demonstrated the safety, efficacy, and feasibility of large-scale manufacturing of the mRNA platform [86]. This success has provided strong momentum for the development of mRNA vaccines against other infectious diseases, including dengue. The basic principle of mRNA vaccines is to encapsulate mRNA sequences encoding target antigens in delivery vehicles such as lipid nanoparticles (LNP). After administration, host–cell ribosomes translate the mRNA into target antigens, thereby activating the immune system and inducing antigen-specific cellular and humoral immune responses [21]. Compared with traditional vaccines, mRNA vaccines offer several advantages, including rapid development timelines, favorable safety profiles, flexible antigen engineering, and simultaneous multi-target immunization. By precisely encoding specific antigen conformations, such as stabilized prefusion forms of the E protein, or by selectively deleting or mutating ADE-associated epitopes, mRNA vaccines may reduce ADE risk at the molecular level. However, mRNA vaccines are not inherently ADE-free, and inappropriate antigen or epitope selection may still induce cross-reactive or subneutralizing antibody responses. In addition, multiple antigens, such as E proteins or non-structural proteins from all four serotypes, can be encoded in a single formulation to achieve multivalent immunity.

### 5.2. Strategies for Addressing ADE Using mRNA Technology

For the central bottleneck in dengue vaccine development, namely ADE, the mRNA platform provides several unique strategies. First, precise antigen engineering can be achieved through rational design of mRNA sequences. Site-directed mutation or deletion of highly conserved but cross-reactivity-prone epitopes, such as the FLE in the E protein, may reduce the generation of non-neutralizing cross-reactive antibodies. At the same time, optimized presentation of serotype-specific neutralizing epitopes such as E-DIII can preferentially induce high-affinity, strongly neutralizing specific antibodies [87]. Second, non-structural protein-targeted strategies may avoid ADE at its source. mRNA vaccines encoding non-structural proteins such as NS3, NS4B, and NS5 can primarily stimulate T-cell immunity without inducing binding antibodies, thereby fundamentally reducing ADE risk. Studies have shown that mRNA vaccines targeting NS3, NS4B, and NS5 successfully induced strong CD8^+^ T-cell responses in transgenic mice [88]. Third, NS1-targeted strategies provide another potential solution. NS1 is a non-structural protein but can be secreted extracellularly. Antibodies against NS1 have a distinct protective mechanism, including the blockade of vascular leakage, and theoretically do not trigger ADE. NS1 mRNA vaccines have shown favorable effects in reducing viremia in animal models [89]. Fourth, multivalent mRNA formulations provide the flexibility to encode antigens from all four DENV serotypes within a single vaccine. Preclinical studies have shown that multi-target mRNA-LNP constructs can induce neutralizing activity against all four serotypes, supporting the feasibility of tetravalent mRNA vaccine design [89]. However, these findings should be regarded as proof-of-concept, and further optimization of the relative expression and immunogenicity of individual serotype components will be required to achieve balanced tetravalent immunity.

### 5.3. Major Preclinical Progress

In recent years, several important preclinical studies have demonstrated the feasibility of using mRNA vaccines against dengue (Table 2).

DENV-2 E80/NS1 mRNA-LNP (2020) [90]: E80 mRNA-LNP, encoding the extracellular domain of the DENV-2 envelope protein without its transmembrane region, elicited high neutralizing antibody titers and provided complete protection against viral infection in mice, but was associated with substantial heterotypic ADE. In contrast, NS1 mRNA vaccination reduced splenic viral loads with a lower level of ADE. These findings suggest that antigen selection may substantially influence the ADE risk of dengue mRNA vaccines. DENV-1 prM/E mRNA-LNP (2021) [88]: The mRNA vaccine encoding the full-length DENV-1 prM/E proteins elicited robust humoral and cellular immune responses in both immunocompetent and immunocompromised mice and protected immunocompromised mice against lethal DENV-1 challenge. Notably, the vaccine induced only minimal cross-reactive enhancement of DENV-2 infection (approximately 1.2-fold), suggesting a lower-than-anticipated risk of antibody-dependent enhancement (ADE). Tetravalent multi-target mRNA-LNP vaccine DENV-ab (2022) [89]: A tetravalent mRNA vaccine targeting NS1 and the envelope protein domain III (E-DIII) was designed to provide coverage against all four DENV serotypes. In mice, the vaccine elicited high levels of neutralizing antibodies against all four serotypes, together with robust T-cell responses. ADE assays showed enhanced infection in fewer than 5% of cells, indicating a favorable safety profile. This study represents one of the most notable advances in tetravalent mRNA-based dengue vaccine development to date and highlights the potential of the mRNA platform to address two major challenges in dengue vaccine development: achieving balanced immunity against all four serotypes while minimizing the risk of ADE. WHO mRNA dengue vaccine R&D consortium (2024): In 2024, the World Health Organization (WHO) promoted the establishment of an international consortium for the research and development of mRNA-based dengue vaccines. Led by the International Vaccine Institute (IVI) in collaboration with institutions including the Chulalongkorn University Vaccine Research Center (Chula VRC), the initiative aims to systematically advance the development of a Target Product Profile (TPP) for mRNA dengue vaccines and to establish a roadmap for their translational and industrial development.

### 5.4. Potential Advantages of Self-Amplifying mRNA Technology

Self-amplifying mRNA (saRNA) is an emerging next-generation mRNA technology. Compared with conventional mRNA vaccines, saRNA employs an embedded RNA-dependent RNA polymerase (RdRp) to enable continuous intracellular self-replication and amplification. As a result, very low doses can produce large amounts of target antigen, which may substantially reduce raw material costs, lower the required vaccine dose, and potentially extend the duration of immunity [91]. This feature is particularly attractive for tetravalent dengue vaccines that must cover all four serotypes. Lower antigen-dose requirements for each serotype may significantly reduce the manufacturing cost of tetravalent formulations and better meet the accessibility needs of developing countries. At present, no saRNA-based dengue vaccine has formally entered clinical trials. Nevertheless, as an important direction for cost reduction and manufacturing scale-up, the saRNA platform has attracted broad attention.

### 5.5. Challenges Facing mRNA Vaccines

Although mRNA vaccines show considerable promise for dengue prevention and control, they still face multiple challenges in practical application. First, current mRNA/LNP formulations still rely heavily on low-temperature storage conditions. However, dengue-endemic regions are largely located in tropical areas with limited resources, where insufficient cold-chain infrastructure constrains vaccine accessibility. Therefore, improving formulation stability at room temperature or under 2–8 °C storage conditions is a key step toward broader application of this technology [92]. Second, mRNA dengue vaccines remain at the preclinical stage and lack long-term human data on immune durability. Sustained protection against all four serotypes is an essential requirement for dengue vaccines. In addition, the reactogenicity of LNP delivery systems and their regulatory effects on immune responses require further optimization. In multivalent vaccine design, expression competition among different antigen-encoding mRNAs may also affect the balance of immune responses. Third, achieving coordinated antigen expression and immune balance among all four serotypes within a single formulation remains one of the core technical challenges. Meanwhile, because dengue primarily affects special populations such as children, the safety and efficacy of mRNA vaccines in these populations require systematic evaluation. Finally, key intellectual property related to mRNA vaccine technology is concentrated among a limited number of companies. This may limit the affordability and accessibility of mRNA dengue vaccines in low-income endemic regions.

Overall, mRNA vaccines provide a new technological pathway for dengue vaccine development. However, further breakthroughs are still needed in formulation stability, immune durability, multivalent design, and global accessibility.

## 6. Future Perspectives and Strategic Recommendations for Dengue Vaccine Development

Based on existing studies and clinical practice, the central challenge in dengue vaccine development lies not solely in antigen selection alone, but rather in how to achieve precise regulation between highly overlapping protective and pathogenic immune responses. The presence of ADE means that the conventional vaccine design concept centered on “enhancing immune responses” is no longer fully applicable to dengue and may even have the opposite effects under certain conditions [93,94]. Therefore, dengue vaccine development is undergoing a paradigm shift from “immune enhancement” toward “immune regulation”.

In this context, the limitations observed in existing live-attenuated and subunit vaccines partly reflect the insufficiency of conventional platforms in controlling antigen expression and directing immune responses. By contrast, mRNA vaccines provide a programmable antigen expression platform, making it possible to selectively present protective epitopes while avoiding potential ADE-associated epitopes. They may also reshape the profile of immune responses through multi-antigen combinations, thereby reducing reliance on a single antibody-mediated mechanism. However, mRNA technology does not inherently guarantee improved safety. It still faces practical challenges in balancing multi-antigen expression, maintaining immune durability, and ensuring accessibility for large-scale application.

Taken together, future dengue vaccine development is expected to gradually shift from experience-driven approaches toward mechanism-guided precision immune design. In addition to the continued optimization of mainstream vaccine platforms, several emerging strategies may offer breakthroughs. These include efficient antigen presentation using nanoparticle-based platforms, induction of cross-serotype T-cell immunity responses through multi-epitope design, and rational antigen optimization through modification of key epitopes [87,95]. Although most of these approaches remain at the preclinical stage, their potential in optimizing antigen presentation, regulating immune bias, and reducing ADE suggests that vaccine design will move toward greater precision and controllability.

At the strategic level, dengue vaccine design should continue to focus on several key issues. First, ADE avoidance should remain a central principle, and antigen structures should be optimized to induce balanced and durable neutralizing immunity against all four serotypes. Second, the clinical translation of emerging platforms such as mRNA vaccines should be accelerated to obtain critical human immunological data. At the same time, research on correlates of protection (CoP) should be strengthened, and controlled human infection models (CHIMs) for dengue should be further developed to improve the efficiency of vaccine evaluation [96]. In addition, improving vaccine stability at room temperature or under standard refrigerated conditions, while enhancing affordability and accessibility through technology transfer and international collaboration are essential prerequisites for achieving global dengue prevention and control goals.

## 7. Conclusions

Dengue vaccine development has undergone decades of exploration. Although licensed vaccines have achieved important progress, key challenges remain unresolved, including ADE, safety in seronegative populations, and imbalanced cross-serotype protection. These challenges fundamentally reflect the complex balance between protective and pathogenic immune responses during DENV infection.

In this context, emerging vaccine technologies represented by mRNA platforms provide new opportunities for dengue vaccine development. Their potential advantages in flexible antigen design, immune-response regulation, and rapid iteration may help drive vaccine development from experience-driven toward mechanism-guided strategies. Although related candidate vaccines remain at the preclinical stage, existing studies have already demonstrated promising results.

Overall, future breakthroughs in dengue vaccine development will depend on the coordinated advancement of immunological mechanism research and engineering innovation, with the ultimate goal of achieving precise regulation of immune responses. Against the background of a continuously increasing global disease burden, accelerating the development of safe, effective, and accessible vaccines is of great importance for dengue prevention and control.

## Figures and Tables

**Table 1:** Major licensed dengue vaccines and vaccine candidates under clinical and preclinical development.

Vaccine/Candidate	Developer	Vaccine Platform/ Technology	Development Status	Key Features/Limitations
Dengvaxia^®^ (CYD-TDV)	Sanofi Pasteur	Tetravalent live-attenuated chimeric vaccine; YF-17D backbone	Licensed in multiple countries	No DENV-derived NS proteins; safety concern in seronegative recipients
Qdenga^®^ (TAK-003)	Takeda	Tetravalent live-attenuated chimeric vaccine; DENV-2 PDK-53 backbone	Licensed in multiple countries	DENV-2-derived NS proteins; serotype-dependent protection
TV003/TV005	NIAID/NIH; licensed to Merck and others	Live-attenuated recombinant vaccine with a chimeric DENV-2 component	Clinical development/research platforml	DENV-derived NS proteins from three serotypes; single-dose platform
Butantan-DV	Instituto Butantan	Live-attenuated recombinant vaccine with a chimeric DENV-2 component	Approved in Brazil	DENV-derived NS proteins from DENV-1, -3, and -4; DENV-3/-4 efficacy remains uncertain
V181	Merck	Live-attenuated recombinant vaccine derived from TV003/TV005	Phase III clinical development	Single-dose candidate; tetravalent efficacy under evaluation
KD-382	KM Biologics	Classical tetravalent live-attenuated vaccine (non-chimeric)	Phase II clinical development	Four serotype-specific attenuated DENV components; clinical efficacy remains to be established
TVDV	WRAIR/U.S. Army	Tetravalent DNA vaccine	Phase I clinical trial	Favorable safety profile; immunogenicity requires further optimization
V180	Merck	Recombinant subunit vaccine	Phase I clinical trial	E-DIII-based subunit vaccine; relatively weak immunogenicity
DENV-ab mRNA-LNP	Academic/research institutions	Tetravalent mRNA vaccine encoding NS1 and E-DIII antigens	Preclinical development (animal studies)	Low potential for ADE; balanced immune responses against all four DENV serotypes; promising development strategy
Tetravalent DENV-VLP	Multiple institutions	Virus-like particle (VLP) vaccine	Preclinical/early-stage development	Noninfectious; prM-depleted design may reduce the risk of ADE

**Table 2:** Representative preclinical studies of mRNA-LNP dengue vaccine candidates.

Vaccine Construct (Reported Year)	Target Antigen (s)	Mouse Model	Main Immune/Protective Response	ADE Assessment
DENV1-NS mRNA-LNP (2019)	DENV-1 NS3/NS4B/NS5	HLA class I transgenic/H-2-null mice (C57BL/6 background)	Strong CD8^+^ T-cell responses; reduced viremia	Not directly assessed
E80 ± NS1 mRNA-LNP (2020)	DENV-2 E80 and NS1	Female BALB/c mice	High neutralizing antibody titers; complete protection	E80-associated ADE; reduced with NS1
prM/E mRNA-LNP (2021)	DENV-1 prM/E	C57BL/6J and AG129 mice	High neutralizing antibody titers; 100% survival after lethal challenge	Low heterotypic ADE
DENV-ab mRNA-LNP (2022)	E-DIII and NS1 from DENV-1–4	Female C57BL/6 mice	Neutralizing responses against all four serotypes; reduced viral burden	No significant ADE detected

## Data Availability

No new data were created or analyzed in this study. Data sharing is not applicable to this article.
